# Upper Airway Epithelial Tissue Transcriptome Analysis Reveals Immune Signatures Associated with COVID-19 Severity in Ghanaians

**DOI:** 10.1155/2024/6668017

**Published:** 2024-02-12

**Authors:** John Demby Sandi, Joshua I. Levy, Kesego Tapela, Mark Zeller, Joshua Afari Yeboah, Daniel Frimpong Saka, Donald S. Grant, Gordon A. Awandare, Peter K. Quashie, Kristian G. Andersen, Lily Paemka

**Affiliations:** ^1^West African Centre for Cell Biology of Infectious Pathogens (WACCBIP), College of Basic and Applied Sciences, University of Ghana, Accra, Ghana; ^2^Department of Biochemistry, Cell and Molecular Biology (BCMB), School of Biological Sciences, College of Basic and Applied Sciences, University of Ghana, Accra, Ghana; ^3^Faculty of Laboratory Medicine, College of Medicine and Allied Health Sciences, University of Sierra Leone, Freetown, Sierra Leone; ^4^Kenema Government Hospital, Kenema, Sierra Leone; ^5^Department of Immunology and Microbiology, The Scripps Research Institute, San Diego, California 92037, USA

## Abstract

The immunological signatures driving the severity of coronavirus disease 19 (COVID-19) in Ghanaians remain poorly understood. We performed bulk transcriptome sequencing of nasopharyngeal samples from severe acute respiratory syndrome coronavirus-2 (SARS-CoV-2)-infected Ghanaians with mild and severe COVID-19, as well as healthy controls to characterize immune signatures at the primary SARS-CoV-2 infection site and identify drivers of disease severity. Generally, a heightened antiviral response was observed in SARS-CoV-2-infected Ghanaians compared with uninfected controls. COVID-19 severity was associated with immune suppression, overexpression of proinflammatory cytokines, including *CRNN*, *IL1A*, *S100A7*, and *IL23A*, and activation of pathways involved in keratinocyte proliferation. *SAMD9L* was among the differentially regulated interferon-stimulated genes in our mild and severe disease cohorts, suggesting that it may play a critical role in SARS-CoV-2 pathogenesis. By comparing our data with a publicly available dataset from a non-African (Indians) (GSE166530), an elevated expression of antiviral response-related genes was noted in COVID-19-infected Ghanaians. Overall, the study describes immune signatures driving COVID-19 severity in Ghanaians and identifies immune drivers that could serve as potential prognostic markers for future outbreaks or pandemics. It further provides important preliminary evidence suggesting differences in antiviral response at the upper respiratory interface in sub-Saharan Africans (Ghanaians) and non-Africans, which could be contributing to the differences in disease outcomes. Further studies using larger datasets from different populations will expand on these findings.

## 1. Introduction

Severe acute respiratory syndrome coronavirus-2 (SARS-CoV-2) emerged to be a significant public health concern driving the ongoing coronavirus disease 19 (COVID-19) pandemic [1]. Beyond the conventional health complications, infection with SARS-CoV-2 was also associated with psychological alterations, including heightened levels of anxiety, stress, and depression, even in hospitalized patients, and this was particularly prevalent during the initial wave of the pandemic [2, 3]. SARS-CoV-2 utilizes the angiotensin-converting enzyme 2 as a receptor for host cell tropism, which is mainly enhanced by the transmembrane protein TMPRSS2 [1, 4]. SARS-CoV-2 infection occurs primarily through the upper respiratory interface, and airway immunity is essential in determining the fate of SARS-CoV-2 infection [5]. COVID-19 is characterized by varying degrees of clinical phenotypes. The majority of SARS-CoV-2 infections remain asymptomatic. Among symptomatic cases, the most common symptoms include fatigue, cough, body pain, weakness, loss of appetite, and fever [6]. About 14%–18% of symptomatic COVID-19 cases progress to a severe clinical phenotype characterized by an aberrant inflammatory response associated with cytokine storm-mediated multiorgan failure and acute respiratory distress syndrome, ultimately leading to COVID-19-associated death [1, 6, 7]. Though other factors may be involved, the differential host gene expression, particularly in relevant tissues, can influence the immune response against infectious pathogens, including SARS-CoV-2. Airway epithelial cells are directly infected by SARS-CoV-2, rendering them essential for identifying immune signatures driving COVID-19 clinical phenotypes. A large body of transcriptomic data describes immune signatures mediating SARS-CoV-2 susceptibility and COVID-19 clinical phenotypes. For instance, using nasopharyngeal swabs (NS), Jain et al. [8] reported a significant association between overexpression of *CCL2*, *CXCL12*, *IL10*, and COVID-19 severity. In a similar study conducted on 36 COVID-19-positive Indian patients, commonly upregulated genes involved in innate immune response were reported [9]. Additionally, marked expression of Th1 chemokines CXCL9/11 and antiviral genes, including *IFIT1* and *OAS* gene isoforms, was associated with enhanced host antiviral response [10]. Generally, all these studies found an association between a compromised antiviral response and uncontrolled inflammatory response mediated by hyperactivation of JAK-STAT, NF-*κ*B, and TGF-*β* signaling pathways through overexpression of proinflammatory cytokines, including *IL6*, *IL10*, *IL23A*, *TNF-α*, and IL18, and COVID-19 severity [8–13]. Though some differences exist due to differences in tissue type, studies have also demonstrated that NS and blood samples share common immune response pathways [14, 15]. Although these studies have shed important insights into SARS-CoV-2 pathophysiology and pathogenic mechanisms, they were primarily conducted in non-Africans. Africans are more genetically diverse than non-Africans, and West Africans, in particular, have a high infectious disease burden [16]. Compared with non-Africans and Black African Americans, marked differences in COVID-19 clinical outcomes were observed in sub-Saharan Africans, particularly West Africans [17–19]. There is currently no publicly available bulk host transcriptomic data from sub-Saharan African populations, especially West Africans. that describe the transcriptome profile at the primary site of SARS-CoV-2 infection. It is, therefore, essential to investigate the differential gene expression in the upper airway epithelial tissue of SARS-CoV-2-infected West Africans underpinning the varying clinical phenotypes.

Ghana is a sub-Saharan African country that reported considerably higher COVID-19 cases among other African countries. Available epidemiologic data reports about 171,600 SARS-CoV-2 infections in Ghana (https://www.afro.who.int/health-topics/coronavirus-covid-19), albeit still lower than seroprevalence studies suggest [20, 21]. Though most of the reported COVID-19 cases are asymptomatic or mild, about 0.9% (1,422) of these infections resulted in COVID-19-associated deaths in Ghanaians. The underlying immunological signatures mediating COVID-19 severity in Ghanaians remain elusive. This study investigated the transcriptomic differences in the upper respiratory interface of SARS-CoV-2-infected Ghanaians with mild and severe clinical phenotypes to characterize immune signatures at the primary SARS-CoV-2 infection site and identify drivers of disease severity. We further compared our data with a publicly available dataset from a SARS-CoV-2-infected non-African population to determine if there are differences in antiviral response.

## 2. Materials and Methods

### 2.1. Study Population

The study population (*n* = 75) included 52 unvaccinated SARS-CoV-2 infected and 23 uninfected Ghanaians from whom NS samples were collected following informed consent at the Ridge Hospital Accra, Ghana. COVID-19-related symptoms accompanied by a positive SAR-CoV-2 polymerase chain reaction (PCR) test were the criteria for inclusion into our COVID-19 disease cohort, while a negative SARS-CoV-2 PCR result and no symptoms of respiratory infection were used as criteria for inclusion as healthy controls. Samples from the SARS-CoV-2-infected individuals were collected at an acute stage of the disease. Clinicians classified COVID-19-infected patients as severe or mild cases according to the disease case definitions. Confirmatory tests for SARS-CoV-2-specific genetic material by real-time reverse transcription-quantitative PCR were performed at the West African Centre for Cell Biology of Infectious Pathogens (WACCBIP), University of Ghana. The clinical record was available only for a few study participants (*Supplementary 1*).

### 2.2. RNA Extraction

RNA was extracted from 300 *µ*l of NS samples using the Quick-RNA Miniprep Plus kit (Zymo Research) following the manufacturer's instructions. Briefly, samples were lysed for 30 min, and nucleic acid was precipitated using absolute ethanol. Sample enrichment for RNA was archived by DNAse treatment followed by column purification. Isolated RNA was eluted in nuclease-free water, and only RNA samples with A260/A280 ratio >1.8 and concentrations above 1 ng/*µ*l were considered for library preparation, as previously examined [22].

### 2.3. Library Preparation and mRNA Sequencing

The NEBNext® Ultra II Directional RNA Library Prep Kit (#7760 L) for Illumina (New England Biolabs) was used for sequencing library construction according to manufacturers' instructions. Briefly, oligo dT-bound beads were used to isolate mRNA, followed by fragmentation for 15 min at 94°C and complementary DNA (cDNA) synthesis. Sequencing libraries were then constructed and amplified using the NEBNext multiple oligos, following manufacturers' instructions. Qubit and TapeStation were used to determine library concentration and size using the high-sensitivity DNA kits. Libraries were generated and sequenced pair-end (150 cycles × 2) on the Illumina Novaseq 6000 system at the Scripps Research Institute using the Novaseq SP reagent kit. Output read files were adapter trimmed and demultiplexed using bcl2fastq v2.20.0.422 (Illumina) to generate unique FASTQ files per sample, with near zero mismatches.

### 2.4. Differential Gene Expression Analysis

FASTQ files were pseudo-aligned to an indexed genome generated from the human cDNA fasta sequence (GRCh38) using Kallisto v0.48.0 [23]. Only samples with >5 million pseudo-aligned human reads (*Supplementary 1*) were used for downstream analysis in RStudio v4.2.1. To control for gender and age in the analysis, the median age of participants, 46.5 years (17–94 years), and *DDX3Y* gene (Y-linked) expression were used to infer participant age and gender, respectively, when absent in the metadata. Transcript IDs were mapped to human genes using an annotated human reference genome (hg38) available in biomaRt v1 [24]. Transcript counts were normalized, and differences in gene expression between groups, while controlling for gender and age, were examined using the likelihood ratio test (lrt) and Wald test (wt) in Sleuth v0.27.3 [25]. The false discovery rate was corrected using the Benjamin–Hochberg test, and gene expression differences with an adjusted *p*-value <0.05 were considered statistically significant. A heatmap of the top differentially expressed genes (DEGs) was generated using the Bioconductor package, ggplot2 version 3.3.6 [26]. Volcano plots were generated using EnhancedVolcano package version 1.14.0 [27], and genes with *p*-value < 0.05 and log2 fold change (log2fc) >1 were reported as upregulated, while those with log2fc <0 were reported as downregulated. GraphPad Prism v9.4.1 was used to construct the violin plots with log-transformed expression values of selected genes, and the significant level was determined using the unpaired *t*-test. ClusterProfiler package v 4.8.2 [28] was used in R version 4.3.1 software for gene set enrichment (GSE) analysis of DEGs to identify associated biological pathways. Pathways with adjust-value < 0.05 were reported.

## 3. Results

After quality control steps, 64 samples (*n* = 64) were analyzed to characterize SARS-CoV-2-induced immune signatures in Ghanaians. Age and gender were self-reported by study participants or, in some cases, by a close relative. The median age of participants, 46.5 years (17–94 years), and *DDX3Y* gene (Y-linked) expression were used to infer participant age and gender, respectively, when absent in the metadata. Females were slightly more represented in the study population at ∼53.1%. Thirty-six individuals (18 males and 18 females) with a median age of 46 years had mild COVID-19. The median age for severe cases in the study population was 79.5 years, and severity was higher in females (4 (66.7%)) compared with men in our study cohort (Table 1). Hypotension was reported in one of the severe cases, while HIV infection, stroke, and hyperglycemia were reported for some individuals with mild COVID-19 for whom clinical records were available (*Supplementary 1*).

### 3.1. Heightened Antiviral Response in the Upper Respiratory Interface of SARS-CoV-2-Infected Ghanaians

**Figure 1:**
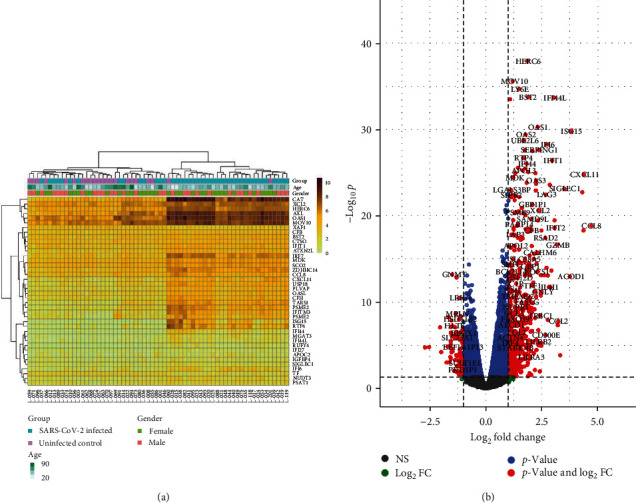
(a) Heatmap of transcript abundance for the top 40 differentially expressed genes in each sample. (b) Volcano plot of upregulated and downregulated genes in SARS-CoV-2 infected Ghanaians compared with uninfected controls. Log2 fold change (FC) cutoff = 1, −Log10 *p*-value.

**Figure 2:**
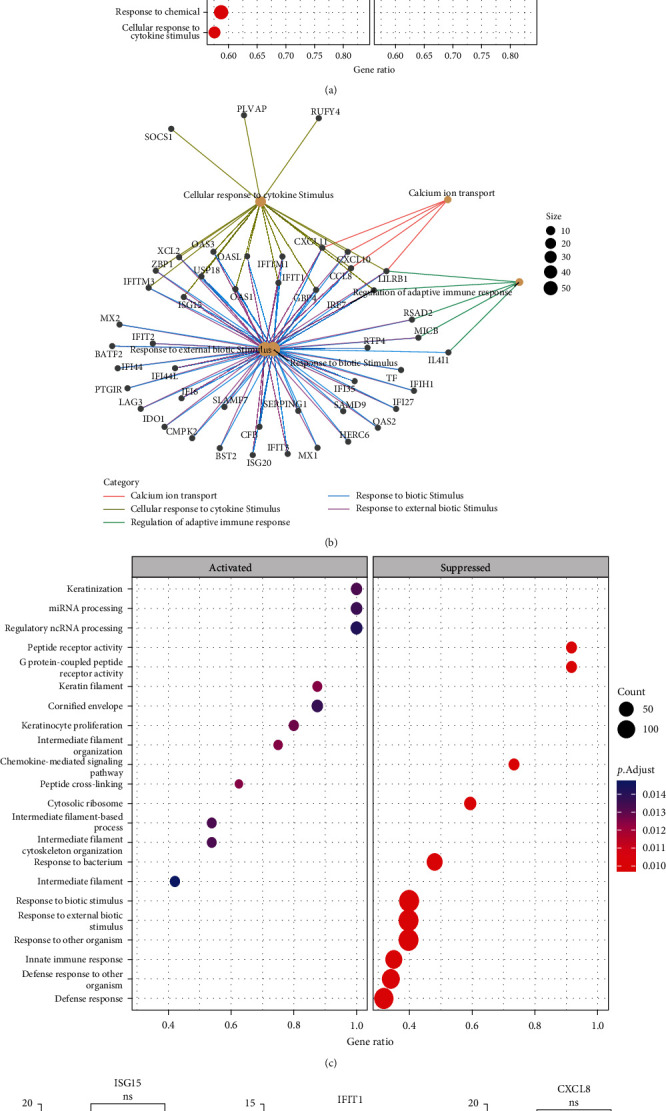
Gene ontology (GO) pathway analysis of top differentially expressed genes in the study cohort. (a) Dotplot showing top activated and suppressed pathways in SARS-CoV-2-infected Ghanaians. Immune response pathways were activated, while cellular biogenesis-related processes were suppressed. (b) Cnetplot showing protein–protein interaction network analysis for the top DEG genes in the COVID-19-infected cohort. (c) Dotplot showing activated and suppressed pathways in severe compared to mild COVID-19 cohorts. Immune response-related pathways or processes were suppressed in individuals with severe COVID-19. Top enriched pathways are shown *p*.adjusted <0.05. (d–f) Violin plots compare the expression of selected antiviral gene expressions in male and female SARS-Cov-2-infected Ghanaians.

To define immune pathways activated during COVID-19 infection in Ghanaians, differences in gene expression in upper respiratory airway epithelial tissue from unvaccinated, uninfected controls and COVID-19-infected Ghanaians (Table 1) were investigated via bulk RNA sequencing of NS. On average, 79% of sequence reads were successfully mapped to the human transcriptome (hg38) (*Supplementary 1*). The likelihood ratio test (lrt) was implemented in the Sleuth package v0.27.3 to identify DEGs [25]. As expected, there was a marked difference in the expression of some immune response genes in the upper respiratory interfaces of COVID-19-infected individuals compared with uninfected controls. We found 1,922 host genes to be differentially expressed in the infected cohort compared with uninfected controls, *q*-value < 0.05, of which 508 and 1,414 were upregulated (log2fc > 1) and downregulated (log2fc < −1), respectively (Figure 1(b), *Supplementary 2* and *3*). Most upregulated genes in the SARS-CoV-2-infected Ghanaian cohort were interferon-stimulated genes (ISGs) such as *BST2, ISG15, OAS1, IRF7, IF16, IFIT1, IFTIM, SAMD9L, CCL8, RSAD2, CCL2, CXCL10*, and *IFI44L* (*Supplementary 2*), known to interfere with viral replication [29–31]. Pathways and processes involved in antiviral immune response, including cytokine-mediated signaling pathway, regulation of adaptive immune response, and immune response process, were significantly activated in the COVID-19-infected cohort (Figure 2(a)), suggestive of a heightened antiviral immune response [29, 32]. There was also evidence of adaptive immune system activation marked by *HLA-A* and *HLA-DR* upregulation (*Supplementary 2*, Figure 2(a)) [32]. In addition to protein-coding genes, the noncoding gene *LGALS17A* was among the top five upregulated genes in SARS-CoV-2-infected Ghanaians. Downregulated genes in our SARS-CoV-2-infected cohort, including *TAF9B*, *TUBA1A*, and *NPBWR1*, are known to be involved in biosynthesis and cellular processes (*Supplementary 3*) [33, 34]. These genes enriched for cellular component biogenesis, which was a significantly downregulated pathway in the COVID-19-infected Cohort (Figure 2(a)), suggesting host cellular function suppression.

The expression of certain ISGs, such as *ISG15*, *IFIT1*, and *CXCL8*, have been reported to be different in males versus females infected with SARS-CoV-2 (*p*-value < 0.05) [35]. By comparing the expression of these genes in our dataset, the difference in their expression in Ghanaian males vs females in our COVID-19 cohort was not statistically significant (Figure 2(d)–2(f)), contrary to a previous report [35].

### 3.2. Impaired Upper-Airway Antiviral Response and Dysregulated Inflammatory Response Mediated by *CRNN* and *IL1A* Overexpression Drive COVID-19 Severity in Ghanaians

**Figure 3:**
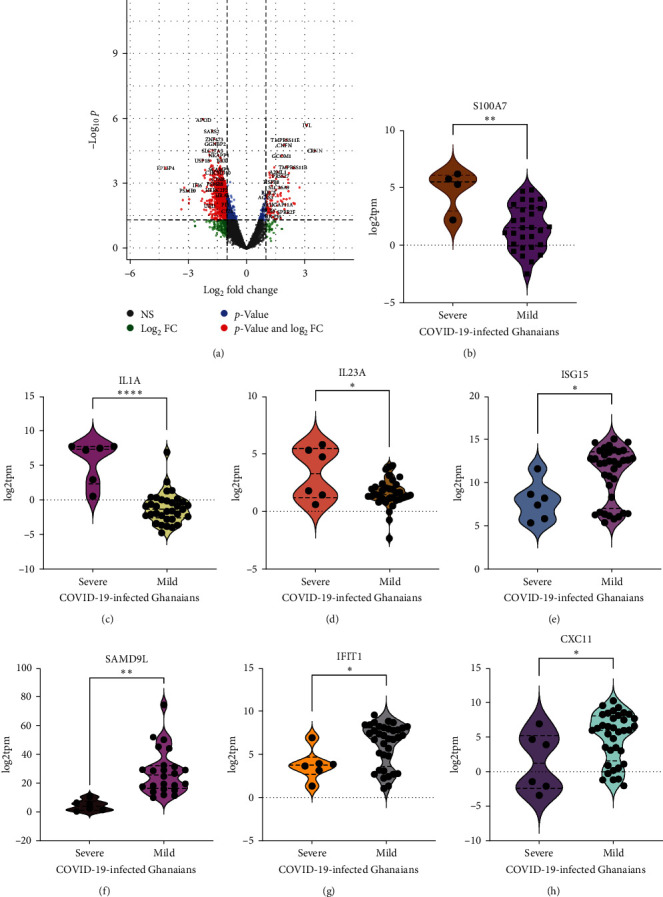
Differentially expressed genes in SARS-CoV-2-infected Ghanaians with severe clinical phenotype compared with mild. (a) Volcano plot showing up- and downregulated genes. *CRNN* was the top overexpressed gene in the severe COVID-19 cohort. (b–d) Violin plots of selected overexpressed proinflammatory cytokines (IL1A, IL23A, and S100A7) in Ghanaians with severe COVID-19. (e–h). Violin plots of selected antiviral-related genes (ISG15, SAMD9L, IFIT1, and CXC11) that were downregulated in severe cases. Log2fc cutoff = 1, −Log10 *p*-value,  ^*∗*^*p*-value < 0.05;  ^*∗∗*^*p*-value < 0.01;  ^*∗∗∗∗*^*p*-value < 0.001.

To identify immune signatures mediating COVID-19 severity in Ghanaians, we compared gene expression differences in the upper respiratory airway of Ghanaians with severe (*n* = 6) and mild (*n* = 36) COVID-19. The median age for severe and mild COVID-19 was 79.5 and 46 years, respectively. Females were more likely to have severe COVID-19 in our study cohort (Table 1). We found 4750 genes to be downregulated (log2fc < −1), while 87 genes were upregulated (log2fc > 1) in individuals with Ghanaians with severe COVID-19 (Figure 3(a), *Supplementary 4* and *5*). Most downregulated genes in the severe COVID-19 cohort, including *ISG15*, *OAS1*, *SAMD9L*, and *IFIT1*, are associated with antiviral response pathways, and immune response-related pathways and processes were suppressed in individuals with severe COVID-19, suggesting an impaired upper-respiratory airway immune response (Figures 2(c) and 3(e)–3(h), *Supplementary 5*). These antiviral-related genes were, however, upregulated in individuals with mild COVID-19, which could explain the immune features underlying the disease's mildness. There was a hyperactivation of keratinization pathways associated with CRNN overexpression [36] and overexpression of proinflammatory cytokines, including *IL23A*, *S100A7*, and *IL1A* (log2fc > 1, *p*-value < 0.05) in Ghanaians with severe COVID-19 compared with mild (Figures 2(c) and 3(a)–3(d). CRNN overexpression has been associated with inflammatory disease [36]. The *MAL* gene, an essential component in NF-*κ*B pathway activation [37], and serine protease *TMPRSS11B* were among the top overexpressed genes in our severe COVID-19 cohort (Figure 3(a), *Supplementary 4*).

Taken together, we found that COVID-19 severity in the Ghanaian cohort was associated with dysregulated inflammatory response mediated by *MAL*, *IL1A*, *IL23A*, *CRNN*, and *S100A7* overexpression and suppression of antiviral immune response-related pathways. A similar association has been reported in other populations [8, 11–13, 38].

### 3.3. Antiviral Genes Are Differentially Expressed in COVID-19-Infected Ghanaians Compared with Non-Africans

**Figure 4:**
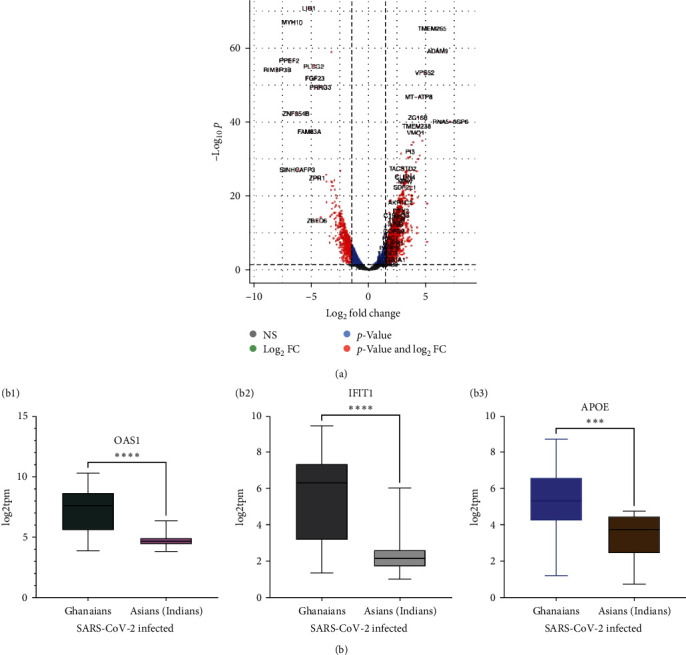
Differentially expressed genes in SARS-CoV-2-infected Ghanaians compared to SARS-CoV-2-infected Indians. (a) Volcano plot showing up and downregulated genes. (b(b1–b3)) Boxplots showing relative expression of selected antiviral genes. Log2 fc cutoff = 1, -Log10 *p*-value,  ^*∗∗∗*^*p*-value < 0.01;  ^*∗∗∗∗*^*p*-value < 0.001.

We further sought to determine whether the expression of antiviral response genes in the upper respiratory interface of SARS-CoV-2-infected Ghanaians differs in other populations. Toward this, we compared our data with a publicly available dataset (GSE166530) from Singh et al. [9] studying COVID-19 immune response signatures in a small cohort of SARS-CoV-2-infected Indians (*n* = 36) within South Telangana, a population characterized by higher COVID-19 severity and mortality [9]. The selection of this data was based on the availability of publicly accessible raw FASTQ data files. Additionally, the data were generated from a similar tissue type, specifically upper airway epithelial tissue, which facilitated a direct comparison. We grouped all cases reported by Singh et al. [9] as a SARS-CoV-2-infected Indian cohort and grouped all the cases from our study to form a SARS-CoV-2-infected Ghanaian cohort. Compared with SARS-CoV-2-infected Indians, an overexpression of antiviral responses-related genes, including *TMEM265, IFI6, ISG15, IFITM3, IFIT1, BST2, CCL2, LCN2*, and *OAS1*, was observed in Ghanaians infected with SARS-CoV-2 (Figures 4(a) and 4(b(1))–4(b(3)), *Supplementary 6* and *7*).

Though preliminary, these observed differences in antiviral gene expression at the primary site of SARS-CoV-2 infection may suggest a more robust innate antiviral immune response in SARS-CoV-2-infected Ghanaians compared to their Indian counterparts. This may have contributed to the reduced COVID-19 severity in Ghanaians and likely other sub-Saharan Africans. Most of these upregulated antiviral genes in SARS-CoV-2-infected Ghanaians were also found to be upregulated in Ghanaians with mild COVID-19 compared to those with severe COVID-19 and uninfected controls (*Supplementary 2*).

## 4. Discussion

The immunological signatures driving COVID-19 severity in Ghanaians remain elusive and need to be better understood. This study investigated the transcriptome differences at the upper respiratory interface of SARS-CoV-2-infected Ghanaians with mild and severe clinical phenotypes to characterize immune signatures at the primary SARS-CoV-2 infection site and identify drivers of disease severity. Consistent with earlier studies [8, 9, 11, 12], we report an upregulation of immune response-related genes accompanied by activation of antiviral pathways and suppression of cellular biogenesis pathways in the upper airway epithelial tissue from COVID-19-infected Ghanaians compared with uninfected controls. *HLA-A* and *HLA-DR* genes were upregulated in the upper airway of SARS-CoV-2-infected Ghanaians (*Supplementary 2*) and are known mediators of the adaptive immune response by antigen processing and presentation [39, 40], suggesting that HLA-A and HLA-DR overexpression may be activating the adaptive immune response vital to virus-infected cell elimination [32]. Cytokines are known regulators of immune response via cell-to-cell communication. Regulation of adaptive immune response was the top enriched activated pathway in our COVID-19-infected cohort compared to controls (Figure 2(a)), suggesting the involvement of cytokines with immune regulatory potential, including *IL-2* [41–43]. In addition to protein-coding genes, non-protein-coding *LGALS17A* was found among the top upregulated genes. Considering the role of noncoding genes in regulating the activities of their target protein-coding genes, *LGALS17A* upregulation may suggest a critical role in SARS-CoV-2 pathophysiology by regulating the activities of a relevant gene(s) involved in SARS-CoV-2 replication. Neuropeptide B/W receptor-1 (*NPBWR1*) is the receptor for Neuropeptides B (NPB) and is required for the activation of NPB/NPBWR1 signaling, which plays a vital role in physiological processes, including energy homeostasis and metabolism [44]. Earlier work has shown that *NPBWR1* knockout mice had defective cellular metabolic processes compared to the wild-type [33, 34]. In this study, *NPBWR1* was among the top downregulated protein-coding genes in our SARS-Cov-2-infected cohort, with cellular component biogenesis being one of the suppressed processes (Figure 2(a)). Noting the critical role of NPBWR1 in metabolic processes to provide the energy and building blocks required for cellular component biogenesis, NPBWR1 downregulation may be driving the suppression of cellular component biogenesis pathways. This could present a previously undescribed SARS-CoV-2 pathogenic mechanism. Comparing Ghanaians with mild vs severe COVID-19 reveals a diminished antiviral response in Ghanaians with severe COVID-19 marked by downregulation of antiviral genes OAS1, *CCL8, SAMD9L, HLA-A, CXCL11, ISG15, IL32*, and *IFIT2*, and suppression of antiviral immune response pathways. A similar trend was also observed in previous studies in other populations [8, 11, 13]. Severe COVID-19 has been chiefly associated with inflammatory cytokines such as interleukin 6 (*IL-6*), *IL-8*, and *IL-10* overexpression [8, 11, 12, 45, 46]. Though Tapela et al. [47] reported some association between *IL-6* and *IL-8* cytokine concentration in plasma samples and COVID-19 severity, the expression of these cytokines was not found to be significantly upregulated in our severe COVID-19 cohort. However, in this study, an upregulation of other pro-inflammatory cytokines, including *CRNN*, *IL1A*, *IL23A*, *IVL*, and *S100A7*, was associated with severe COVID-19. *CRNN* was the most upregulated gene, and keratinization was the top-activated process in individuals with severe COVID-19 cohort. Keratinocytes represent the first line of the host defense system, and their hyperproliferation contributes to the pathogenesis by infiltration of inflammatory cells [48, 49]. CRNN overexpression was previously shown to aberrantly regulate keratinization by activating the Phosphoinositide 3-Kinase/Akt Pathway, leading to inflammatory diseases, such as psoriasis [36]. Epithelial cells are directly infected during SARS-CoV-2; thus, CRNN overexpression in our severe COVID-19 cohort may represent a potential pathogenic mechanism employed by SARS-CoV-2 to induce dysregulated inflammatory response via upregulating keratinization at the primary site of infection. In addition, the *MAL* gene, an important component in NF-*κ*B signaling pathway activation [37], and TMPRSS11B were among the top 10 upregulated genes in Ghanaians with severe COVID-19. TMPRSS11B is implicated as a driver of lung carcinoma [50], and severe COVID-19 is associated with lung abnormalities [51, 52]. Since SARS-CoV-2 is known to induce pathology in the lung, *TMPRSS11B* upregulation in individuals with severe COVID-19 may also represent another SARS-CoV-2 pathogenic mechanism. *TMPRSS11B* also interacts with *CRNN* (*Supplementary 1*). Our result on immune signatures mediating COVID-19 severity in Ghanaians agrees substantially with findings from other studies [8, 11, 12, 46, 53]. The *SAMD9L* pathway was previously shown to be a critical host barrier that poxviruses subvert most to establish an infection [54] and was among the ISGs found to be significantly downregulated in Ghanaians with severe COVID-19 compared with mild cases. The suppression of SAMD9L in individuals with severe COVID-19 suggests that it may also be a critical host restriction factor that SARS-CoV-2 must antagonize to establish disease. Additionally, *MUC21*, a gene previously associated with lung adenocarcinoma, was also upregulated in Ghanaians with severe COVID-19 [55]. We found an insignificant difference in the expression of previously reported antiviral genes, *ISG15*, *IFIT1*, and *CXCL8*, in males and females Ghanaians infected with SARS-CoV-2, contrary to a previous report [35]. However, this observation might be influenced by the sample size used in this study (Table 1).

COVID-19 severity is considerably lower in sub-Saharan Africans, particularly West Africans, compared with non-Africans and Black African Americans [16, 21, 56]. We observe an upregulation of genes involved in antiviral response pathways, including OAS1 that mediates RNase L pathway [57, 58], IFIT1, and APOE at the upper respiratory airway of COVID-19-infected Ghanaians compared with a relevant publicly available dataset (GSE166530) from an Indian COVID-19 cohort [9] (Figure 4). The upregulation of these antiviral genes in COVID-19-infected Ghanaians may suggest a more robust antiviral response at this critical interface. Though preliminary, this observed difference in antiviral gene expression at primary infection sites may have contributed to the reduced COVID-19 severity in sub-Saharan Africans, particularly Ghanaians. To our knowledge, this is the first direct comparison of immune response-related gene expression in the upper respiratory interface between SARS-CoV-2-infected West Africans and a non-African population and the first COVID-19 bulk host transcriptome dataset from West Africans.

## 5. Conclusions

In conclusion, this study describes immune signatures at the primary site of SARS-CoV-2 infection and identifies immune signatures driving COVID-19 severity in SARS-CoV-2-infected Ghanaians. It further provides important preliminary evidence suggesting that antiviral genes are more highly expressed at the primary site of SARS-CoV-2 infection in sub-Saharan Africans (Ghanaians) compared with non-Africans (Indians), which may be driving the differences in antiviral response and clinical outcomes. Our overall report on DEGs in COVID-19-infected Ghanaians corroborates previous reports from similar studies.

## Figures and Tables

**Table 1:** Disease characteristics of COVID-19-infected Ghanaians used in this study.

Participants characteristics	All participants (*N* = 64)	COVID-19 cases (*N* = 42)		Uninfected control (*N* = 22)
		Mild (*N* = 36)	Severe (*N* = 6)	
Female	34 (53.1%)	18 (50%)	4 (66.7%)	12 (54.5%)
Male	30 (46.9%)	18 (50%)	2 (33.3%)	10 (45.5%)
Age (median)	46.5 (years)	46 (years)	79.5 (years)	49.7 (years)
Symptoms				
Fever	—	31 (86.1%)	6 (100%)	1 (4.5%)
Cough	—	27 (75%)	6 (100%)	0
Shortness of breath	—	0	6 (100%)	0
Headache	—	36 (100%)	4 (66.7%)	2 (9.1%)
Running nose	—	32 (88.9%)	5 (83.3%)	0
Sore throat	—	19 (52.8%)	5 (83.3%)	0
Fatigue	—	9 (25%)	6 (100%)	0
Muscle and joint pain	—	13 (36.1%)	6 (100%)	0
Chill	—	0	6 (100%)	0
Required mechanical ventilation	—	0	2 (33.3%)	0

Shortness of breath, chills, and mechanical ventilation were associated with severity.

## Data Availability

Processed data are available in the Gene Expression Omnibus (GEO) database with accession number GSE215906.

## Additional Points

*Limitations*. First, the proportion of individuals with severe COVID-19 was small (*n* = 6) compared with the mild (*n* = 36). Additionally, records on comorbid conditions for most of the participants were unavailable. This was partly due to the challenge of obtaining clinical records during pandemics. Nevertheless, since our findings are generally consistent with previous studies in other populations, we can confidently assume that there were no major comorbidities in our cohort that could have significantly impacted our results. Future bulk transcriptome profiling studies using airway epithelial tissue from a much larger COVID-19-infected Ghanaian cohort with established clinical records would strengthen and extend this work.

## Supplementary Materials

Supplementary 1 Processed data.

Supplementary 2 Up sars-CoV-2-infected vs uninfected control Ghanaians.

Supplementary 3 Down sars-CoV-2-infected vs uninfected control Ghanaians.

Supplementary 4 Up COVID-19-severe vs mild Ghanaians.

Supplementary 5 Down COVID-19-severe vs mild Ghanaians.

Supplementary 6 Up infected-Ghanaians vs Indians.

Supplementary 7 Down infected-Ghanaians vs Indians.

Supplementary 8 Figure 1: Protein-Protein interaction of top differentially expressed genes.
