# Supplementary figures and images for: Upper Airway Epithelial Tissue Transcriptome Analysis Reveals Immune Signatures Associated with COVID-19 Severity in Ghanaians
Source: J Immunol Res. 2024 Feb 12;2024:6668017. doi: 10.1155/2024/6668017 (PMC10876312; doi:10.1155/2024/6668017)

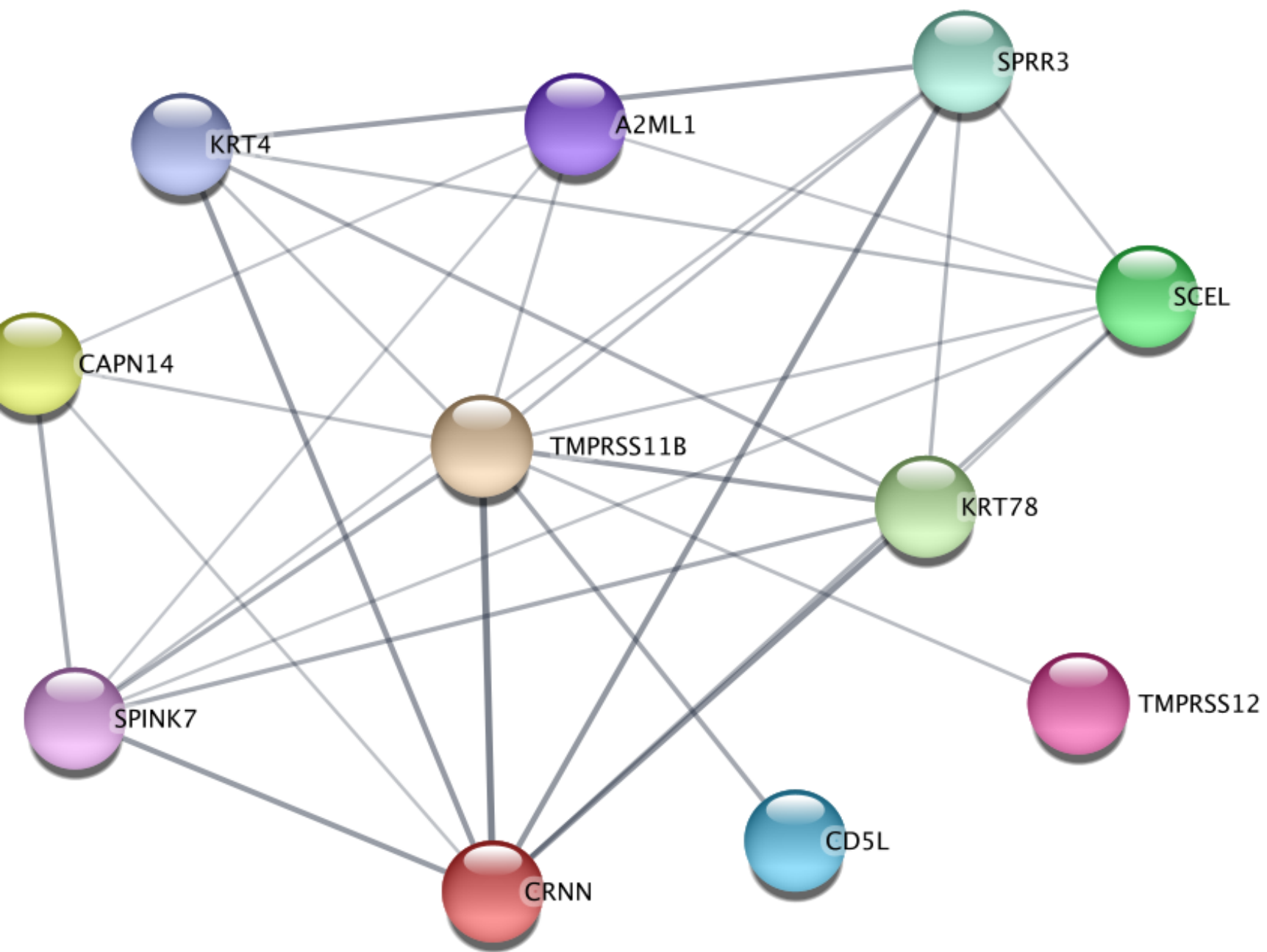

Supplement: Supplementary 8 — Figure 1: Protein-Protein interaction of top differentially expressed genes. [file 6668017.f8.pdf]
